# Antibacterial nanocomposite of chitosan/silver nanocrystals/graphene oxide (ChAgG) development for its potential use in bioactive wound dressings

**DOI:** 10.1038/s41598-023-29015-y

**Published:** 2023-06-23

**Authors:** Yoxkin Estévez-Martínez, Rubí Vázquez Mora, Yesica Itzel Méndez Ramírez, Elizabeth Chavira-Martínez, Rafael Huirache-Acuña, Jorge Noé Díaz-de-León-Hernández, Luis Jesús Villarreal-Gómez

**Affiliations:** 1 Tecnológico Nacional de México, Campús Acatlán de Osorio, Unidad Tecnológica Acatlán, Carretera Acatlán-San Juan Ixcaquistla kilómetro 5.5, Del Maestro, 74949 Acatlán, Puebla Mexico; 2 Instituto de Investigaciones en Materiales, Universidad Nacional Autónoma de México, Circuito Escolar S/N, Ciudad Universitaria, 04510 Ciudad de México, Mexico; 3 Facultad de Ingeniería Química, Universidad Michoacana de San Nicolás de Hidalgo, 58060 Morelia, Michoacán Mexico; 4 Centro de Nanociencias y Nanotecnología, Universidad Nacional Autónoma de México, Carretera Tijuana-Ensenada, Km. 107, 22860 Ensenada, Baja California Mexico; 5 Facultad de Ciencias de la Ingeniería y Tecnología, Universidad Autónoma de Baja California, Unidad Valle de las Palmas, Blvd. Universitario #1000, CP 21500 Tijuana, Baja California Mexico; 6 Facultad de Ciencias Química e Ingeniería, Universidad Autónoma de Baja California, UABC, Parque Internacional Industrial Tijuana, Universidad #14418, 22424 Tijuana, Baja California Mexico

**Keywords:** Structural materials, Materials science, Nanoscale materials, Graphene, Nanoparticles, Structural properties, Synthesis and processing

## Abstract

An adequate wound dressing reduces time of healing, provides cost-effective care, thereby improving patients’ quality life. An antimicrobial bioactivity is always desired, for that reason, the objective of this work is to design an antimicrobial nanocomposite of chitosan/silver nanocrystals/graphene oxide (ChAgG). ChAgG nanostructured composite material is composed of chitosan from corn (Ch), and silver nanocrystals from garlic (*Allium sativum*). The nanocomposite obtained is the result of a series of experiments combining the graphene oxide (GrOx) with two members of the *Amaryllidaceae* family; garlic and onion (*Allium cebae*), which contain different sulfur materials. The characterization arrays confirmed the successful production of silver crystal, graphene oxidation and the blending of both components. The role of the chitosan as a binder between graphene and silver nanocrystals is proved. Moreover, the study discusses garlic as an optimal source that permits the synthesis of silver nanocrystals (AgNCs) (⁓ 2 to 10 nm) with better thermal and crystallinity properties. It was also confirmed the successful production of the ChAgG nanocomposite. *Escherichia coli* and *Staphylococcus aureus* were used to demonstrate the antibacterial bioactivity and L-929 fibroblast cells were utilized to visualize their biocompatibility. The proposed ChAgG nanomaterial will be useful for functionalizing specific fiber network that represents current challenging research in the fabrication of bioactive wound dressings.

## Introduction

Burn wounds are one of the main causes of skin damage. Based on World Health Organization statistics, almost 300,000 people worldwide die from burns each year. In severe burns, the affected cells and blood vessels are often injured and the blood supply to the wound is disturbed. Many factors such as oxygenation, infection, aging, hormones, and nutrition can potentially influence burn progression and disrupt repair with an unbalanced release of various growth factors and cytokines. Different treatment approaches such as dressings and skin substitutes have been applied to aid wound healing. A thorough understanding of the effective factors of burns can improve wound healing outcomes^1^.

The main purpose of wound dressing is to generate a temporary protective physical barrier, absorb wound drainage, and provide the required moisture to optimize re-epithelialization. The choice of dressing depends on the anatomical and pathophysiological characteristics of the wound. Contemporary wound dressings provide additional benefits, such as antimicrobial properties and pain relief^2^.

Despite all, the main issue to solve is bacterial infections in chronic wounds. Electrospun nanofibers offer a promising solution to the management of wound healing, and numerous options are available to load antibacterial compounds onto the nanofiber webs^3^.

The development of new and/or improved antibacterial compound formulations with alternative mechanisms of action is taken into consideration as an important topic to include them in a wound dressing design for overcoming antimicrobial resistance^4^.

In 2021, Su et al.^5^ demonstrated a strong antimicrobial capacity of a chitosan/silver nanoparticle/graphene oxide nanocomposite using both Gram-positive (*Staphylococcus aureus*) and Gram-negative (*Escherichia coli*) bacteria. The authors discussed that combining chitosan with graphene oxide promotes interesting properties for its proposal in a wide range of applications, especially in the biomedical field.

Silver nanostructures such as nanocrystals, nanofibers, or nanocrystals possess antimicrobial activity which is useful for its loading in drug delivery systems such as wound dressings and topical gels to treat local wounds^6^. On the other hand, different carbon nanostructures have been proposed to be used in biomedical applications, mainly graphene^6–10^. Currently, different reviews in the literature have reflected these studies, mainly in films of chitosan compounds with oxidized graphene^11^.

The blend of chitosan, silver nanocrystals, and graphene has been previously investigated demonstrating a great capacity for biomedical applications^12^. Chitosan is biodegradable, non-toxic, and promotes wound healing^13^. Silver on nanometer scales provides antimicrobial properties and reduces inflammation^14^, and graphene presents antibacterial properties on its own or mixed with different substances. Besides, it has excellent mechanical properties, conductivity, and biocompatibility^7^.

Hence, this work aims to prepare and characterize a blend nanocomposite of chitosan, AgNCs, and graphene (ChAgG) and validate its potential biological characteristics to functionalize electrospun fibers for wound dressings. A physicochemical characterization is presented through simultaneous Thermal Analyzer DSC/TGA (SDT), X-ray diffraction (XRD), scanning electron microscopy (SEM), high-resolution transmission electron microscopy (HRTEM), Raman spectroscopy and X-ray photoelectron spectroscopy (XPS) demonstrating the nanocomposite conjugation. To demonstrate the potential use for its incorporation in an electrospun wound dressing, an antibacterial test using *Escherichia coli* and *Staphylococcus aureus* was performed. Finally, a cytotoxicity biocompatibility test using L-929 fibroblast cells were used.

## Materials and methods

### Materials

The antimicrobial nanocomposite ChAgG was prepared using chitosan (*Ch*) (Merck). Graphene powders (*Gr*) (LP Bond Research and Development of the Third Millennium S.A. of C.V), and *AgNO*_*3*_ (*OMNICHEM*) were used as received. Acetic acid at 1% v/v and *H*_*2*_*O*_*2*_ at 30% v/v were used as solvents (Merck). For the synthesis of the nanocrystals (AgNCs), *AgNO*_*3*_ from the commercial company OMNICHEM was used. *NH*_*4*_*OH,* distilled water, *Allium sativum* extract (garlic), and *Allium cebae* (onion) were obtained in the central market of the City of Acatlán de Osorio, México.

### Graphene oxidation (GrOx)

Once the graphene powders (*Gr*) were shredded in a food processor at 600 W for 30 s, they were dispersed at a concentration of 2.41% w/v in hydrogen peroxide (*H*_*2*_*O*_*2*_) at 30% v/v for a period of 30 s. Subsequently, this mixture was placed in a sonic bath at a frequency of 1 Hz for 25 min, until the mixture was homogeneous. The mixture was then placed in a conventional microwave oven for 30 s. After that, the container was removed and placed again in a sonic bath at 1 Hz for 60 s. These last steps were repeated 10 times. Completing these cycles of reactions by microwaves and sonic baths, the temperature increases, and a boiling graphene solution is observed, because of that, it was allowed to stand for 3–4 h cooling to an ambient temperature. To neutralize the *H*_*2*_*O*_*2*_, isopropanol was added in a 1:1 v/v ratio with respect of the *H*_*2*_*O*_*2*_. Finally, a filtering system which includes a Kitasato, Büchner funnel, and a 0.20 µm diameter nitrocellulose membrane was set up to bring the solid residues detained in the membrane, the filtered samples were dried in a conventional oven (FELISA brand) (± 5 °C) at 100 °C obtaining dry graphene. It was stored and labeled in an amber-colored vial until its use.

### Chitosan solution (Ch)

A 2% w/v chitosan solution (Ch), from Merck, was prepared using 1% v/v acetic acid (*CH*_3_–*COOH*). To obtain a homogeneous solution, the solution was blended in a food processor at 600 Watts for 30 s and then left to stand at room temperature until there are no more bubbles on the surface.

### Synthesis of silver nanocrystals (AgNCs)

In order to obtain a higher yield in the synthesis of silver nanocrystals in our ChAgG nanocomposite, in this research, a series of experiments was performed to find the one with the highest yield, that is, choosing between garlic extracts (*Allium sativum*) (A1–A5) and onion extracts (*Allium cebae*) (C1–C5), taking into account the base experience by some authors of this research group in the patent previously reported^15^.

Therefore, an experimental design was created where different variables were combined, such as the use of the plant extracts in water (*H*_*2*_*O*) or in ammonium hydroxide (*NH*_*4*_*OH*), using silver nitrate (*AgNO*_*3*_) in crystal (solid) or in solution (liquid), maintaining in both cases a concentration of 0.98 mmol. To avoid confusing the reader, the TGA and XRD characterization of the samples with the highest concentration of silver nanocrystals between garlic and onion extracts, as well as the HRTEM of the silver nanocrystals used in the ChAgG nanocomposite are presented, where the A5 sample was selected for the synthesis of the ChAgG nanocomposite.

The garlic and onion extracts used for this study were obtained in the Mixtec lowland region of Puebla; specifically in the central market of the City of Acatlán de Osorio, Puebla State of México Republic; located geographically in the coordinates of latitude 18° 21′ 30″ and longitude − 98.048611 West with respect to the meridian of Greenwich and 1260 m. s. n. m., with an average temperature of 24 °C.

## Synthesis of silver nanocrystals (AgNCs) and incorporation of graphene and chitosan solution (Ch)

Based on the patent application for the synthesis of AgNCs^15^, garlic and onion were crushed in bi-distilled water at a concentration of 10.9% w /v for 30 s in a food processor at 600 Watts. The mixture was left to stand for 12 h, changing from a color yellow tone to turquoise blue. Sulfur compounds, such as alliin, (+)-S-methyl-l-cysteine ​​sulfoxide, and γ-l-glutamyl-S-allyl-l-cysteine ​​from garlic were extracted^16^ as well as thiopropanal sulfoxide, dipropenyl disulfides and propenyl propyl disulfides were obtained from onion^17^. At the end of this period, the organic solid residues were separated through a cloth mesh by vacuum filtration, and a reflux unit was used (heating and stirring rack, flask, and refrigerant) to bring this garlic solution to a temperature of 75 °C. Once this temperature was reached, 1 mmol of *AgNO*_*3*_ was added for 30 min. A color change from turquoise blue to a brick-brown color is observed in the solution, indicating the formation of silver nanocrystals^18^. After 30 min of reaction at reflux conditions, the oxidized graphene was added in a 2:1 m/m ratio to the AgNCs’s solution, at the same temperature of 75 °C for another 30 min. After this time, the chitosan solution was added at 2% w/v, with an initial concentration of 0.6% v/v to the entire solution, maintaining the temperature reaction at 75 °C for the last 30 min. Finally, a filtering system with Kitasato, Büchner funnel, and 0.20 µm diameter nitrocellulose membrane was assembled and used to obtain the corresponding solids, thus comprising the formation of the graphene nanocomposite, silver nanocrystals, and chitosan (ChAgG compound).

### Characterization

#### Simultaneous differential scanning calorimetry, DSC, and thermogravimetric analysis, TGA (SDT)

SDT was carried out with a high-resolution TA Instruments 2950 thermogravimetric analyzer. Samples were evaluated in a temperature range from room temperature to 500 °C at a heating rate of 10 °C/ min in air. To perform this analysis of each of the samples, only 2 mL of each sample was taken in liquid, evaporation was not performed because it was possible to obtain results in the liquid state. The liquid samples were stored in a refrigerator (− 4 °C) before being analyzed.

#### X-ray powder diffraction (XRD)

The purity of the reagent and the *Ag* crystal structure was confirmed by powder X-ray diffraction (XRD) in a Bruker AXS Model D-B Advance. At room temperature from the range of 5° to 70° for A1 and A5 samples, start: 2.000°–end: 70.000° for ChAgG sample—Step: 0.020°–Step time: 1.2 s–Time Started: 8 s–*Cu* radiation. The nanocrystals that were infiltrated in the garlic, were subjected to a temperature of 95 °C for 8 days. in an oven (± 5 °C) to perform the evaporation of the solvents and water, and later perform this analysis because of the detachment of the nanocrystals in each of the crystallizers. Any problem was identified at the laboratory temperature and humidity conditions. For the nanocrystals embedded in the onion, the temperature at which they were initially subjected was the same used for garlic, but the detachment of the nanocrystals was not achieved from the crystallizer; so, each of the samples of the onion was subjected to a temperature of 250 °C for 5 days. to achieve detachment of the onion and garlic of the nanocrystals and with this, the unit analysis of the nanocrystals can be performed to determine the crystal structure and morphology.

#### Electron microscopy

**Figure 1:**
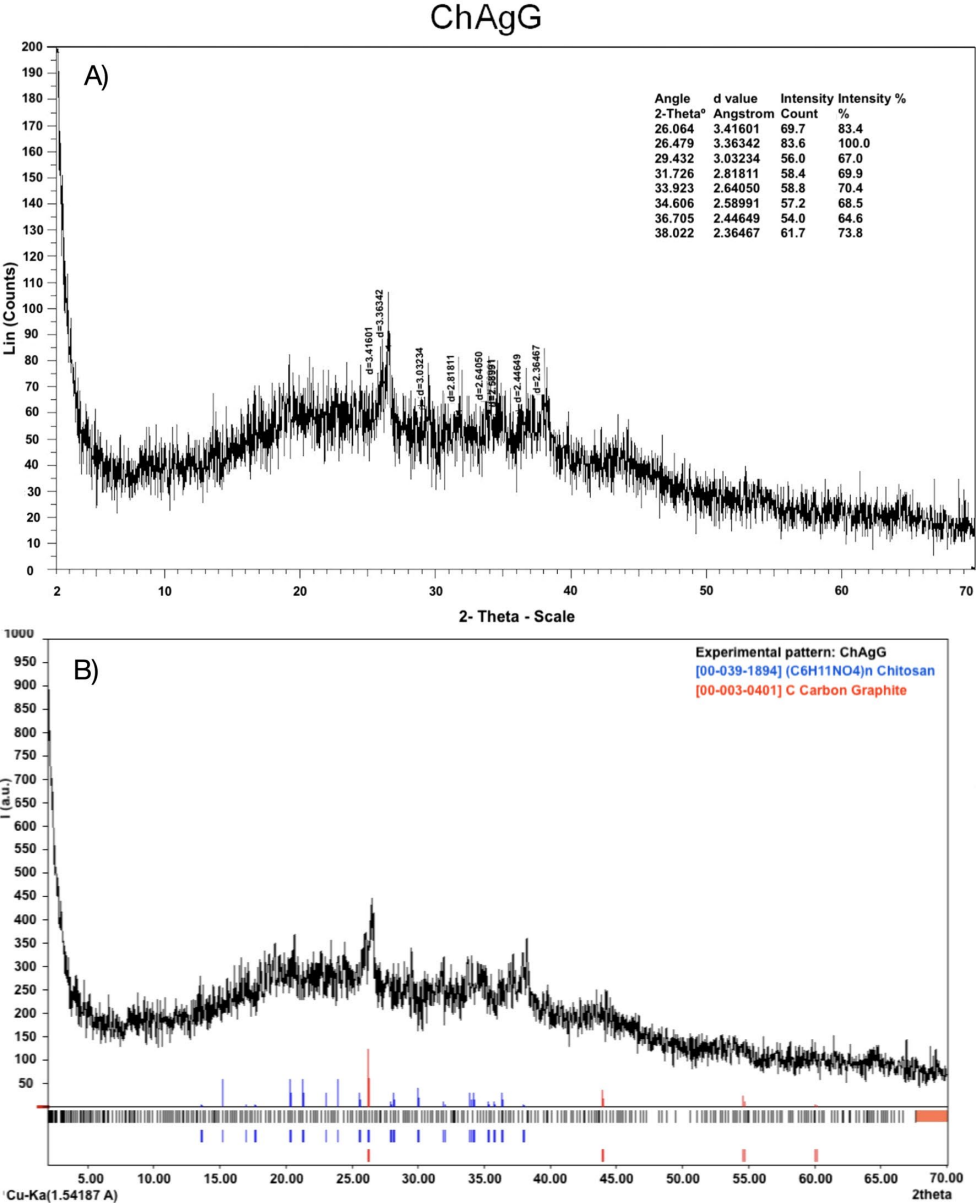
XRD for the sample corresponding to ChAgG nanocomposite.

A verification of the nanocrystal morphology on garlic (*Allium sativum*) was performed by Scanning Electron Microscopy (SEM), on a Leica Stereoscan 440, and High-Resolution Transmission Electron Microscopy (HRTEM) on JEOL equipment model JEM-1200EX. The analysis by SEM required that samples of garlic (*Allium sativum*) and onion (*Allium cebae*) were subjected to a solvent evaporation process in an oven at a temperature of 95 °C, for 5 days and then the nanocrystals were removed from the containers (crystallizers). For this analysis HRTEM, the garlic (*Allium sativum*) and onion (*Allium cebae*) samples were subjected to the same treatments as in the previous analyzes, but for better evaporation of the solvents, the temperature was adjusted at 400 °C for 3 h. To assemble the samples, a solution with nanocrystals in methanol was made by 10 min shaking in an ultrasound bath. A count of the size of 50 nanocrystals presents in the SEM figure field (50 nm) and 30 nanograins conglomerates in the HRTEM figure (1 µm) were measured, and an average particle <  = change to grain diameters and standard deviations were calculated using the Image J^®^ software.

#### Raman spectrometry

The Raman spectrometer was equipped with a 532 nm laser and a CCD detector. The detector temperature was kept below 5 °C and a filter of 0.3 was set for the laser. An × 50 objective was used for all measurements, which means a 1.4 × 100 multiplication of the image size. Confocal analysis was performed with a spatial resolution of 1 × 1 μm, which is within the size of a graphite particle (10 μm for the material under study).

#### X-ray photoelectron spectrometry (XPS)

The XPS apparatus also possesses a μ-FOCUS 500 X-ray monochromator (Al, A5 and ChAgG excitation line). Binding energies were referenced to the adventitious C1s, O1s, and Ag peaks. Background subtraction was performed using a Shirley baseline, while mixed Gaussian/Lorentzian functions were used to fit core-level spectra.

#### Antibacterial test

ChAgG sample powder was dissolved in 0.1% dimethyl sulfoxide (DMSO) solvent at a concentration of 10 mg/mL. The antimicrobial activity test was performed using the serial dilution method^19^ and carried out by inoculating the pathogenic strains *Escherichia coli* (ATCC 25922) and *Staphylococcus aureus* (ATCC 25923), previously incubated for 24 h at 35 °C in Mueller–Hinton medium (MHB at 23 g/L) in a 96 microwell plate, where the inoculated medium was placed, adding the sample at a concentration of 10 mg/mL in 0.1% DMSO solvent.

Controls (gentamicin as a positive control and inoculated MHB culture medium as negative control), were handled in parallel to verify the inhibition and normal growth of the pathogens. After the inoculation was completed, the absorbance of each well was read at 620 nm using a microplate spectrophotometer (Thermo Scientific Multiskan). Subsequently, the microplates were incubated at 35° for 24, 48, and 72 h, in order to observe the inhibitory effect on the strains of the samples.

A volume of 96 μL of MHB medium was inoculated with the pathogen strain to be tested and deposited in row A of the microplate, to which 4 μL of the sample (at a concentration of 10 mg/mL in 0.1% DMSO solvent) was added and homogenized. From rows B to H, a volume of 50 μL of inoculated MHB medium was deposited. A volume of 50 μL of the homogenate from row A was taken and transferred to row B by homogenizing and completing a total volume of 100 μL, of which a volume of 50 μL was taken, repeating this procedure consecutively until reaching the wells of row H, from which the remaining 50 μL were discarded. Eight dilutions of the sample of interest were obtained from the dilution process (400, 200, 100, 50, 25, 12.5, 6.25, and 3.125 µg/mL), and incubated at 35° for 24, 48, and 72 h, after which the absorbance of the microwell plate was measured at 492 nm, to calculate the inhibitory effect for each of the established concentrations. An inhibition curve was constructed at 24, 48, and 72 h of exposure, separately calculating dose response at these times, which would establish the minimum inhibitory concentration (MIC) for each sample. All experiments were done in triplicate. Statistical analysis (ANOVA P > 0.05) was performed for all groups.

#### MTT cell viability tests

The prepared ChAgG sample (10 mg/mL 0.1% DMSO solvent), was also used for the MTT cell viability test. ATCC CCL-1, fibroblast cell line NCTC Clone 929 [L cell, L-929, derivative of Strain L] from *mus musculus* tissue, were used for culturing. The cells were cultivated in DMEM, supplemented with 10% fetal bovine serum (FBS) and 100 U/mL penicillin–streptomycin, at 37 °C in 5% CO_2_, until they reached the desired confluency (80%). ChAgG samples were prepared using the eight dilutions (400, 200, 100, 50, 25, 12.5, 6.25, and 3.125 µg/mL) prepared for the antibacterial test. Then, 10, 000 cells were added per well of the L-929 fibroblasts exposing for 24 h to the ChAgG samples under the same conditions of cell incubation. Where diluted phenol 10 mg/mL was used as the positive control and the negative control was the cell suspension without any treatment.

After time passed, 10 µL MTT solutions to the 100 µL of the medium in each well were added. The plate was mixed by tapping gently on the side of the tray. Then, it was incubated at 37 °C for 4 h. Subsequently, 200 µL DMSO directly into the medium in each well was added and pipetted up and down several times to dissolve the formazan salt. Finally, the measure of the absorbance signal was made on a spectrophotometer at 570/630 nm. Experiments were done in triplicate. Average absorbance and standard deviation were calculated. Statistical analysis (ANOVA P > 0.05) was performed for all groups.

## Results and discussions

### TGA of silver nanocrystals (AgNCs)

Despite there are several characterization techniques for the determination of the presence of nanometric crystals such as Transmission Electron Microscopy (TEM), X-ray Diffraction (XRD), Atomic Force Microscopy (AFM), Dynamic Laser Scattering (DLS), Small-Angle X-ray Scattering (SAXS), amongst others^20^, TGA also provides important information about the thermal stability and kinetic information of the compounds adding evidence of the incorporation of the silver nanocrystals (AgNCs) to the sample^21^. The obtention of silver nanostructures can be seen by thermal analysis such as TGA, on account of the high thermal resistance of silver nanocrystals. Despite changes in morphology and size that can be observed with the increment of temperature, silver nanocrystals remain unaltered at high temperatures. When silver nanocrystals are synthesized using a biological source, it is easy to detect because organic matter degrades around 100 °C^22^.

It can be seen at the Supplementary Information (Figure-S1) of TGA that a process of mass loss begins at 90% mass percentage and 100 °C. It can be concluded that it is because of the degradation of the organic matter present in the samples, since water starts evaporating at those temperature conditions, (96 °C), like the *NH*_*4*_*OH*^23,24^ (38–58 °C) and *AgNO*_3_ where its operating temperature shift (212–444 °C) due to the presence of all materials. In Figure-S1A around 240 °C the beginning of a small curve is observed, indicating the total decomposition of the reagent. Given that in Figure-S1B the same behavior is not observed, as a consequence of the onion kinetics.

According to what it is seen in Figure-S1, it was observed that there is the change in the reaction kinetics of garlic and onion, owing to the observed residual mass of ~ 5% present in samples A1(Figure-S1C) and A5 (Figure-S1D) which are still *AgNO*_3_ reagent present at ~ 250 °C, this observed residual mass represented the presence of silver nanocrystals only seen in the garlic source. Hence, it was decided to continue the further synthesis of the silver nanocrystals from the garlic sourceand discard the onion source due to the total discharge of *AgNO*_*3*_, significant residual mass that can compete with the alternative source (garlic).

Adebayo-Tayo et al. also use the thermogravimetric analysis to monitor the bioproduction of silver nanocrystals using *Oscillatoria *sp., extract, where they observed a residual mass of 23.46% that was not degraded even at higher temperatures (884–94 °C), endorsing that SDT can be used to demonstrate the presence of silver structures^25^.

#### X-ray diffraction (XRD) of silver nanocrystals (AgNCs)

As described in the methodology section, the crystallinity of the silver nanocrystals synthesized from garlic and onion, were compared using the X-Ray Diffraction (XRD) technique. AgNCs samples obtained from garlic presented a higher crystallinity and number of nanocrystals, being A1 and A5 samples the ones with the best results (In Supplementary Information Figure-S2).

The graph shows signs which are assigned to A1 and A5 experiments in which more nanocrystals were obtained. In the garlic samples, the presence in the diffraction peaks at 2θ = 38.2° and 44.2° mainly represent crystal planes of Ag, corresponding to (111) and (200)^26^, in another reference, 2θ = 38.1◦ and 44.3◦ corresponding to Ag (111) and Ag (200)^27^.

The characterization of the nanocrystals presented in the samples with *AgNO*_*3*_ and different solvents was carried out using the X-ray diffraction (XRD) technique. The graph shows signs which are assigned to A1 and A5 experiments in which more nanocrystals were obtained. In the garlic samples, the presence of AgCl as chlorargyrite (PDF 00-001-1238) was observed, presenting a crystalline cubic structure (Figure-S2) with a = 5.54910 Å, b = 5.54910 Å, c = 5.54910 Å, alpha = 90,000, beta = 90,000, gamma = 90,000 binding energies that confirm the structure and presence of silver.

With this characterization, we can observe that sample A5 is the one that has a higher crystallinity with respect to A1^21^. For this reason, it is the ideal one to be used for the synthesis of the nanostructured ChAgG composite.

### X-ray diffraction (XRD) of nancomposite (ChAgG)

ChAgG observed in Fig. 1A XRD data, the d values 5.8300, 4.3700, 4.1700 and 4.1700 repeated correspond to Chitosan with PDF 00-039-1894 interest characteristic that presents four reflexions (Fig. 1B in blue lines) of 100% intensity corresponding to (*hkl*) planes: (120), (102), (022) and (200). Additionally, in red lines Carbon identifying through PDF 00-003-0401, exhibited in d values 3.4000, 2.0600 and 1.6800. As expected, AgNCs are not observed, since they are less than 3%. As it is known to all^28^ the DRX can detect the materials present in the range of 1–3%, depending on the material analyzed. Continuing with the analysis of the diffractogram as expected, the amorphous part that comprises the nanostructured, organic material and sample holder is observed like a very wide reflection.

In another perspective in the diffractogram of the nanocomposite ChAgG, in Fig. 1B, the blue lines correspond to Chitosan with PDF 00-039-1894. Additionally, Carbon identifying through PDF 00-003-0401, exhibited in red lines.

### SEM of silver nanocrystals (AgNCs)

**Figure 2:**
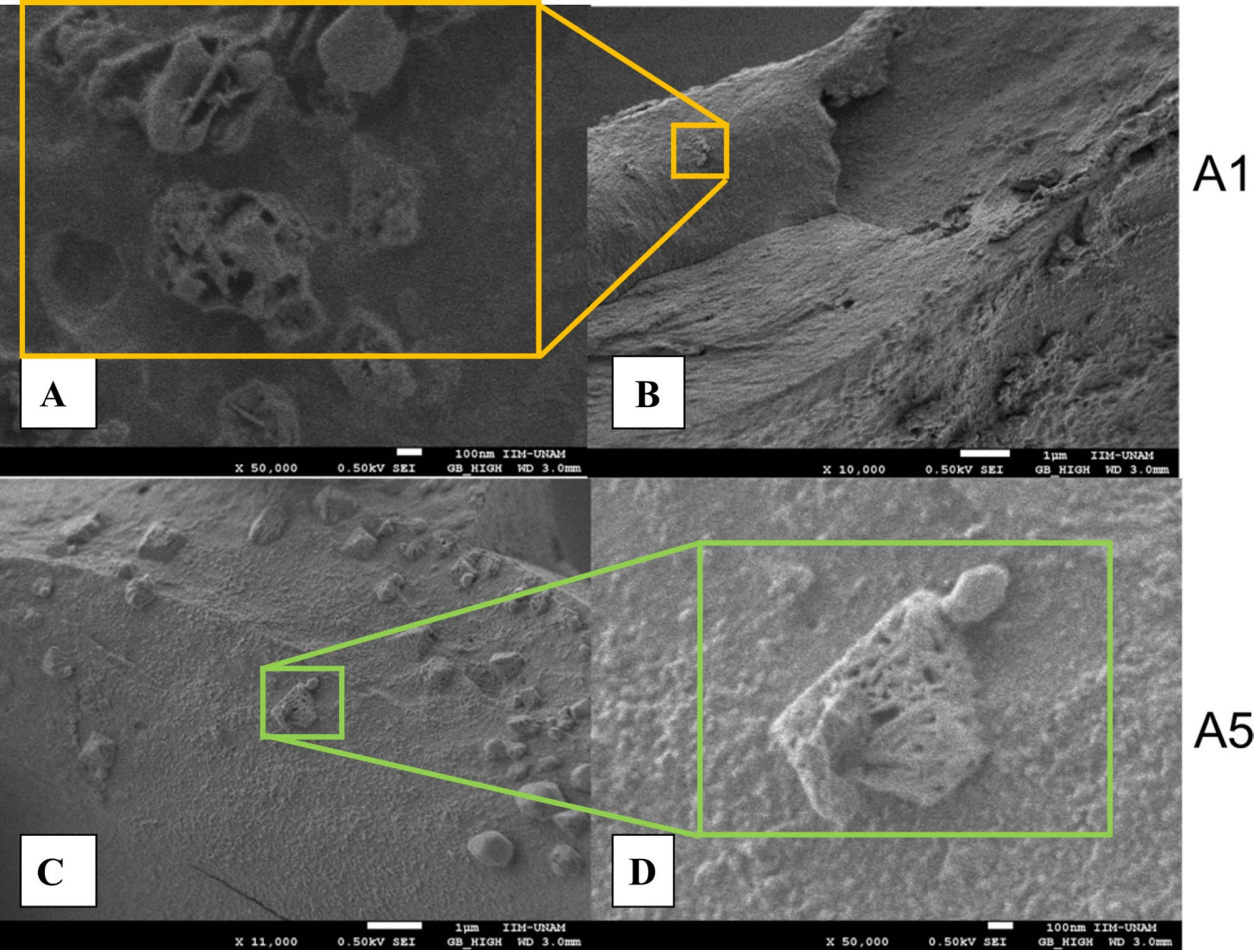
SEM for nanocrystals obtained from garlic (Allium Sativum). (**A**) A1 sample with 100 nm (×50,000) amplification; (**B**) A1 sample with 1 µm (×10,000) amplification; (**C**) A5 sample with 1 µm (×11,000) amplification; (**D**) A5 sample with 100 nm (×50,000) amplification.

Figure 2 shows the AgNCs obtained for the garlic source. These samples show mostly crystalline cubic conglomerates of ~ 100 and 200 nm which can contain silver nanocrystals deposited over residual organic material from the garlic source. The calculated conglomerate nanograin average diameter and standard deviation after the measuring of 30 crystals in the field were 384.6** ± **168.2 nm (Fig. 2C). It can be said that crystalline conglomerates are in turn made up of much smaller nanocrystals, which, for the resolution of their size, cannot be determined by this technique. Figure 2A is an amplified image of Fig. 2B where an irregular shape porous crystals can be appreciated from sample A1. On the contrary, images from sample A5 look mostly as a cubic crystalline morphology with a better definition. In Fig. 2C, several nanocomposites can be observed that were used to calculate the average diameter and standard deviation. Finally, Fig. 2D showed a more crystalline-like cubic, rectangular form with certain porosity and irregularities on the surface.

Vijayakumar et al.^29^, formulated silver nanocrystals from garlic clove as a main source. HR-TEM images demonstrated the presence of cubic and spherical-shaped crystals and are uniformly dispersed. The particle size was measured resulting in between ~ 10 and 50 nm.

Su et al.^5^ prepared a chitosan/silver nanoparticle/graphene oxide nanocomposite using an atmospheric pressure microplasma process. In that study, TEM results showed the morphology and size distribution of the nanocomposites with an average size of ~ 73.69 nm. In these compounds, two groups of samples were prepared (composited with or without graphene oxide). Samples without graphene oxide showed that the morphology of the AgNCs appears to be mostly spherical, and that they have smaller particle sizes (mean particle size ~ 27.5 nm), and are better dispersed than a group of samples with the presence of graphene oxide where the AgNCs appear to be more agglomerated (raspberry-like) and have a larger particle size (mean particle size ~ 73.69 nm).

In another study, silver nanocrystals (AgNCs) using 4-amino-5-hydroxynaphthalene-2,7-disulphonic acid monosodium salt (AHNDMS) and functionalized with p-Aminobenzoic acid (PABA) were prepared, as a sensitive and selective colorimetric sensor for nitrite ions. In that study, SEM results were described, the resulted AgNCs have spherical shape, but, when nitrite ion was introduced with certain concentration to the functionalized AgNCs, the spherical AgNCs changed to an aggregated one, indicating that the presence of the NO^2−^ in the sensor induced the aggregation of the functionalized AgNCs by selective diazotization coupling reaction in the nanoparticle system^30^.

Moreover, in a study which a green synthesized AgNCs from *Nigella sativa* leaf extract was compared with other chemical synthesis methods for AgNCs. The research group examined their samples trough SEM and particle size analysis that revealed that most of green synthesized AgNCs were spherical in shape with the average size ~ 15 nm and are often agglomerated into small aggregates, while comparing with chemically-synthesized nanocrystals predominately were spherical in shape with the average size 30 nm and were often agglomerated into large aggregates (more than ~ 500 nm)^31^.

In our study, nanocomposite sizes were bigger than in the previously mentioned studies, and this can be reflected in the antimicrobial properties observed, compared with the cited references, despite those studies which evaluated the antimicrobial bioactivity, different methods than ours were performed, reporting excellent antimicrobial properties. In our study, we do not have excellent results in both bacteria, but for Gram-negative bacteria (*E. coli*) excellent bioactivity was demonstrated contrary to the *S. aureus* strain. It is well known that the antimicrobial properties of silver nanocrystals are related to size, whereas lower sizes have better antimicrobial properties^32^. Despite that, less cytotoxicity in our samples was demonstrated compared with the above studies at similar nanocomposite concentrations.

### HRTEM of silver nanocrystals (AgNCs)

**Figure 3:**
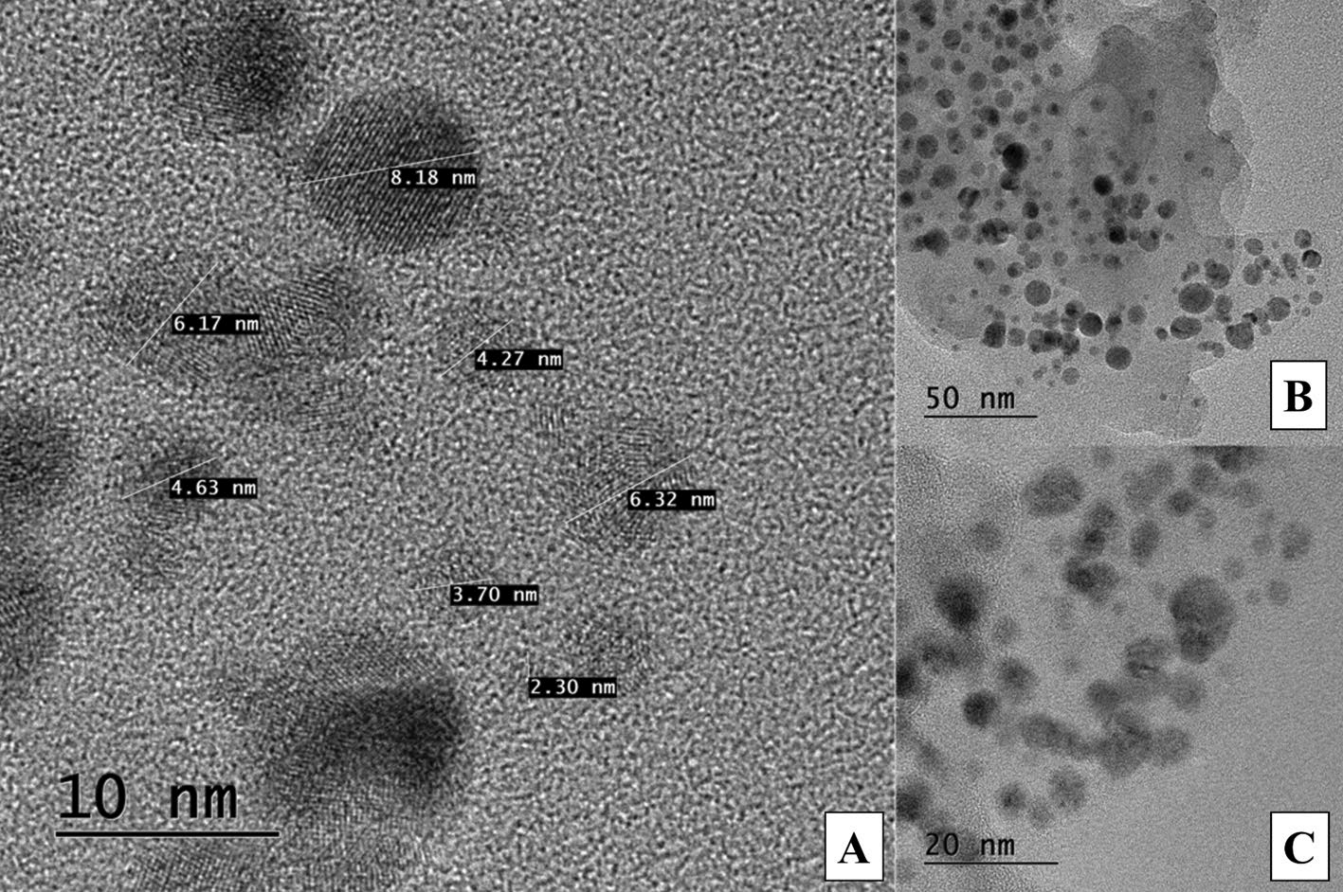
High-resolution transmission electron micrographs for nanocrystals obtained from a garlic sample (*Allium cebae*). (**A**) with the highest amplification (10 nm measuring bar); (**B**) with the lowest amplification (50 nm measuring bar); (**C**) with the medium amplification (20 nm measuring bar).

The morphology of metallic AgNCs was observed using high-resolution transmission electron microscopy (Fig. 3). Figure 3A,C, shows the obtained products (AgNCs) resulting from their synthesis using 1 mmol *AgNO*_*3*_ in polycrystals 0.0425 mg (A5), the sample used on the ChAgG nanocomposite. The presented micrograph shows the cubic morphology of the obtained nanocrystals in form of crystals, which have diameters in the order of ~ 2 to 10 nm. The calculated nanoparticle average diameter and standard deviation after the measuring of 50 crystals in the field were 5.6 ± 1.5 nm (Fig. 3B).

Regarding the identification of crystalline phase, space group and orientation of the NPs related to the d-spacing of the lattice fringes reported in the HRTEM image were shown in this study, because in the XRD the crystalline structure of the AgNCs through AgCl minerals such as chlorargyrite are described and discussed in the above section.

It was found that within the same batch of nanocrystals, the arrangements can be fitted to the crystallinity of the supercrystals (3D assemblies) in face center cubic (fcc), hexagonal close-packed (hcp) and body center cubic (bcc) structures and random compact packaging (rcp). This is due to the collective property, which is due to dipolar interactions or to the order itself (intrinsic), according to what is reported in the literature^33,34^.

In this study, the spectroscopic characterization (XPS, XRD, and Raman) gives evidence of the covalent and ionic interaction of the AgNCs with the oxygen atoms of the oxidized graphene and chitosan discussed in the mentioned sections, these AgNCs are shown in the HRTEM (Fig. 3A).

Khan et al.^35^ prepared through a rapid green synthesis AgNCs using curcumin. In that study, HRTEM was used with the unique purpose in calculating the size of the crystalline nanocrystals, recorded as 12.6 ± 3.8 nm (doubled higher size than our study), which was confirmed by HRTEM, while the face-centered cubic (fcc) crystallographic structure was confirmed by powder x-ray diffraction (PXRD) and selected area (electron) diffraction SAED^35^.

In another study, Chung et al.^36^ discussed the green synthesis of AgNCs using plant sources such as *Piper longum, Manilkara zapota, Prosopis juliflora, and Codium capitatum extracts,* amongst others. The applications of these AgNCs are intended to be used in biomedical applications such as cancer treatment. As same as our study and Khan’s group^35^, the HRTEM and SEM were used to observe the morphology and calculate the average sizes of AgNCs (1–100 nm), and crystallographic structure characteristics were analyzed by the XRD^36^.

Comparing the obtained results with the reported literature which synthesized AgNCs from plants, smaller particle sizes but with agglomerated cubic structures were obtained. The resulted nanostructured material presented excellent bioactive properties^32,36^.

HRTEM morphology of AgNCs (8.18 nm from Fig. 3A) with enlarged lattice fringes, fast Fourier transform (FFT) and the inverse FFT (IFFT) patterns, and IFFT profile with spacing value d for a specific plane (Supplementary Information Figure-S3). Analysis of d-spacing values was carried out using the Gatan Digital Micrograph Software application, which resulted in d_hkl_ values of 0.235 nm and 0.2439 nm for a set of crystal planes at different sites on the AgNCs (8.18 nm of diameter) surface. HRTEM micrographs exhibited well-defined lattice fringes of d_111_Ag plane (Figure-S3B) confirming the clear crystalline nature of prepared AgNCs^37–39^.

The XRD pattern of AgNCs presented earlier in Figure-S2, revealed 2 major peaks corresponding to (111) and (200) planes of fcc structure, corresponding to Ag (111) and Ag (200)^27^ (AgCl as chlorargyrite (PDF 00–001-1238). The d-spacing values of the derived diffraction planes from d_111_Ag = 0.2428 nm and d_200_Ag = 0.2126 nm, are in good agreement with XRD diffraction pattern of AgNCs^39^.

### Raman of oxidized graphene and ChAgG nanocomposite

The strongest and most representative bands in the Raman study of carbon structures are at 1580 and 1362 cm^−1^, attributed to the G band and the disorder-induced D band, respectively, analogous to those of graphite. On the other hand, the Raman characteristics of graphene-based materials depend not only on the phonon properties but also on the corresponding electronic properties, for example, the linear band structure in single-layer graphene (1LG) and the structures of the different bands. In 1LG, the origin of the harmonic and combining modes, like the 2D modes, define a triple resonance Raman scattering (TRRS) process, which is related to the linear dispersion of its electronic bands. In other words, by keeping this 2D band in mind, we have multilayer graphene, and it appears around ∼ 2700 cm^−1^^40^. The G band (1580 cm^−1^) corresponds to vibrations in the graphite plane with the phonon modes in the E2g symmetry (longitudinal optics (LO) and transverse optics (TO) in the plane). Therefore, the maximum position of the G-mode is sensitive to external perturbations, such as defects, doping, strain, and temperature, and is therefore widely used to probe the responses of graphene-based materials and related devices to external perturbations. In the high-frequency region, due to the weak interlayer coupling in Multilayer Graphene (MLG), the maximum position of the G mode, Pos (G), in the intrinsic N-layer graphene (NLG) is almost 1582 cm^−1^ and it is not sensitive as such to the number of layers of graphene (N). However, in graphene structures with less than ten layers, they show parameters that depend significantly on the number of layers they have in graphene (N). The D band (1362 cm^−1^) is generally associated with the respiratory vibrations of the sp2 rings. Therefore, this band is associated with the different disorders that the different NLGs have, which exhibit similar spectral characteristics to the 2D mode. The corresponding fundamental modes of the 2D and D peaks require a surface defect for their activation in Raman scattering. Thus, the absence of the 2D band would give us evidence of the existence of a graphene monolayer of high quality (1LG)^41^.

**Figure 4:**
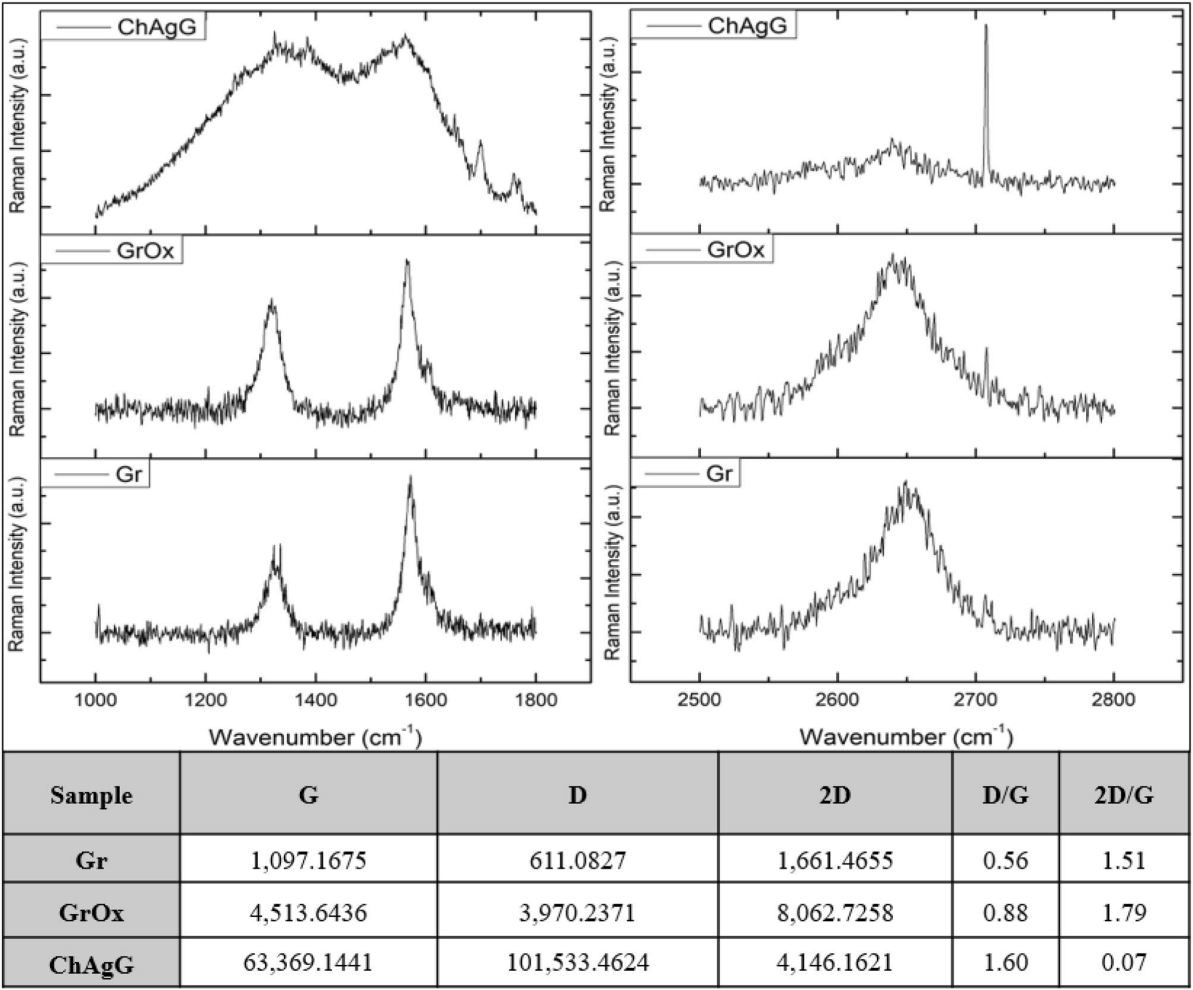
Raman spectroscopy of graphene (Gr), oxidized graphene (GrOx) and the nanocomposite (ChAgG).

According to the results obtained and the tabulated values (Fig. 4) ​​of D, G, and 2D, with respect to what has been explained above, it was observed that these spectra allow us to identify the signals corresponding to graphene nanostructures characteristics^42^. On the other hand, to identify the characteristic bands of chitosan and silver nanocrystals, these are indicated based on what is cited in the literature, which indicates that chitosan can be found at 2885 cm^−1^ and 1654 cm^−1^^43^, mainly the amine group of chitosan at 1593 cm^−1^^44^. While AgNCs are concerned, as previously reported, where these nanostructures have decorated chitosan in aerogel, they showed an important interaction in the range of 1000–1800 cm^−1^^45^, precisely where the ChAgG spectrum widens enormously, overlapping between them, the signals are interacting, corresponding to the D and G bands of graphene, chitosan and silver nanocrystals. This broadening has also been documented when silver nanocrystals interact with graphene bilayers^46^.

According to the symmetrical shape of the signal identified as 2D, it can be deduced that there is a graphene monolayer, with respect to the 2D/G signal ratio. It can also be concluded that graphene does not have important structural defects until it interacts with AgNCs. However, the increase in the D/G ratio makes it possible to identify surface defects in the oxidation and, at the same time, a disturbance in its lamellar structure when interacting with the silver nanocrystals and chitosan, which when the graphene is oxidized, these oxides are at the extremes of the sheets and that lead to the grafting between the silver nanocrystals and the graphene sheets. Chitosan plays as a binder for the stability of these two nanostructures^47^.

### X-ray photoelectron spectroscopy (XPS) of oxidized graphene and ChAgG nanocomposite

**Figure 5:**
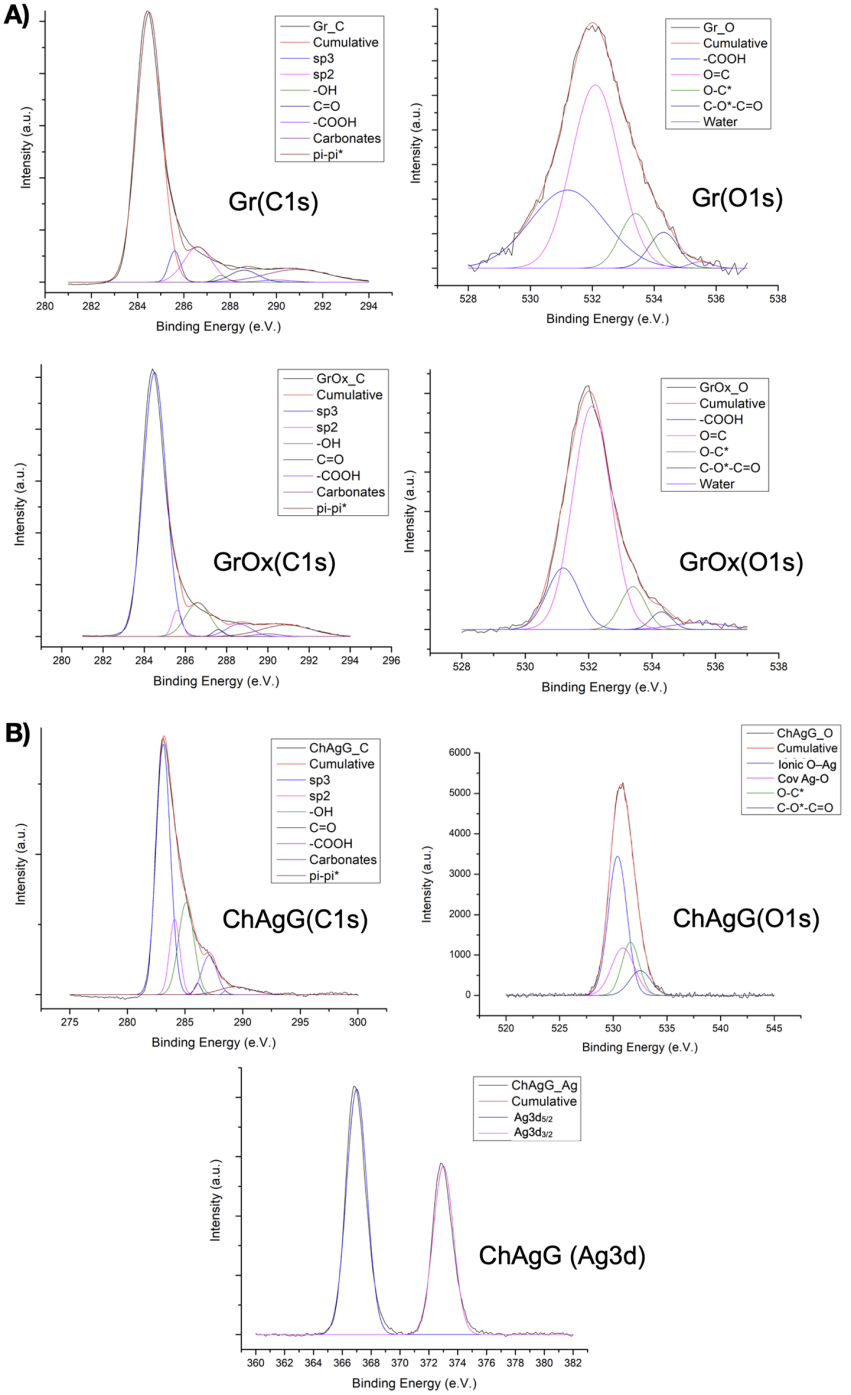
(**A**) XPS deconvolution of C1s and O1s of the Gr and GrOx. (**B**) XPS deconvolution of C1s and O1s of the Nanocomposite ChAgG (Ag3d).

XPS was used to identify the presence of AgNCs in the ChAgG nanocomposite with respect to carbon and oxygen. The XPS spectra of graphene (Gr) and oxidized graphene (GrOx), were recorded as a reference. The range of binding energy for carbon (*C1s*) is at 295–281 eV, for oxygen (*O*1*s*) is at 540–528 eV and for silver is at 364–376 eV. The three band ranges were evaluated for the nanocomposite ChAgG and, the reference (Gr and GrOx) only *C1s* and *O1s*. The deconvolution of the *C*1*s* and *O*1*s* peaks was based on the common data of different reports on XPS^48–51^. For Gr and GrOx (Fig. 5A), the *C*1*s* peak is at 284.5 eV for *sp*^2^ hybridization, at 285.6 eV for *sp*^3^ hybridization, at 286.6 eV when oxidized graphene has hydroxyl groups (*–OH*), at 287.6 eV for carbonyl (*C*=*O*), at 288.6 eV for carboxyl groups (*–COOH*) of oxidized graphene, at 290 eV for carbonate (C–O*–C=*O*) and, at 290.9 eV for pi–pi* (π–π*) electronic transitions characteristics in graphene sheets. O1s peaks at 531.2, 532.1, 533.4, 534.3, and 535.5 eV for the molecules of carboxyl (*–COOH*), carbonyl (*O*=*C*), hydroxyl (*O–C*), carbonate (C–O*–C=*O*) and water (*H*_*2*_*O)*, respectively.

The nanocomposite ChAgG peaks (Fig. 5B) are presented. In *C1s*, they are the same deconvoluted bands, to compare the presence of the characteristic peaks of graphene in *C1s* with the nanocomposite. Although, for the *O1s* band, the silver–oxygen peaks are considered, where the XPS shows a range of 528–535 eV, as it has been studied since the late 1990s^52^ and which is still cited today^53^. 530.4, 530.9, 531.6, and 532.5 eV for the molecules ionic bond (O–Ag), covalent bond (Ag–O), hydroxyl (*O–C*), and carbonate (C–O*–C=*O*), respectively. Finally, there are the two bands that are fingerprints, about the presence of the AgNCs^26,53^, this refers to the binding energies of Ag3d_5/2_ and Ag3d_3/2_ were 367 eV and 373 eV, respectively.

Analyzing the percentage arrangement of the peaks described in C1s of the three samples (Supplementary Information Figure-S4), it can be observed that the sp2 hybridization in the nanocomposite decreases considerably, close to 20% with respect to the Gr sample, while when graphene is oxidized (GrOx), this value increases about 2%, as a consequence of the small exfoliation that exists when they are oxidized, according to studies that have been carried out by the authors of this publication^51^. On the other hand, we can also notice that the hybridization of the defects (sp3) rises with respect to the references, as well as the –OH and carboxyl^54^. The increase of the π–π* electronic transitions (at 290.9 eV) in oxidized graphene compared to only graphene, suggests that there is exfoliation of the graphene sheets due to peroxide^55^.

On the other hand, when analyzing the percentage arrangement of the peaks described in O1s of the reference (Gr and GrOx), Supplementary Information Figure-S5, it is visible that when graphene is oxidized, the carboxyl groups decrease, while the carbonyls increase, while in the other molecules, the proportion remains imperceptible.

To describe the O1s peak of the ChAgG nanocomposite, Supplementary Information Figure-S6, based on the cited reference^26,53^, It is observed that 50% belongs to an ionic interaction between oxygen-silver, while 21% of this band corresponds to a covalent interaction between these atoms, deducing that the nanocrystals are linked by these two means to oxidized graphene and to molecules of chitosan.

In this study, the Raman and XPS of the chitosan and AgNCs were not performed, since with respect to the Raman assay, only the D/G, and 2D/G bands of the graphene were studied, which are considered fingerprints to demonstrate the grafting on the surface of our nanocomposite. Neither, the XPS of the AgNCs nor chitosan were necessary, because the objective of the XPS is to obtain the presence of silver and its interaction with the oxygen molecules, either from oxidized graphene or of chitosan, for which there is evidence and it has been reported.

In this case, it can be said with certainty that the presence of the AgNCs is interacting with the oxygen atoms of the oxidized graphene and chitosan, which together with the other structural characterizations (XRD, TEM, XPS), we have a nanocomposite of graphene, chitosan and silver nanocrystals, called ChAgG.

## Biological characterization

### Antibacterial tests

Gram-negative *Escherichia coli* (ATCC 25922) and Gram-positive *Staphylococcus aureus* (ATCC 25923) bacteria were selected to evaluate the antibacterial activity of ChAgG samples. In general, the bioactivity of the ChAgG sample differs from the two bacteria tested representing each of their differences in cell wall composition. Nevertheless, the results showed that the ChAgG sample decrease the bacterial population of both bacteria using the highest concentration (400 µg/mL). Still, ChAgG bioactivity was more effective in *Escherichia coli* (ATCC 25922) than its counterpart *Staphylococcus aureus* (ATCC 25923).

In the case of the *Staphylococcus aureus* (ATCC 25923) exposed to ChAgG samples no important reduction (ANOVA P > 0.05) of cell growth was observed, a greater decrease was seen with concentrations of 400 and 200 µg/mL, where approximately a ~ 20% of cell growth reduction was showed. The highest bioactivity observed was with a concentration of 200 µg/mL at 48 h, and 12.5 µg/mL at 72 h, with ~ 75% of cell growth reported. At the lowest concentrations such as 6.25 and 3.125 µg/mL, no alteration in cell proliferation was detected at any time. These results allow us to conclude that, in general, the ChAgG samples were not effective against *Staphylococcus aureus* (ATCC 25923).

In contrast, Table 1 summarizes the ChAgG samples that were in contact with *Escherichia coli* (ATCC 25922) showed a more notable antibacterial effect than in the study with *Staphylococcus aureus* (ATCC 25923), whereas with just 3.125 µg/mL of the ChAgG we observed a pretty interesting bacterial bioactivity reducing bacterial population about ~ 52, 54 and 62% at 24, 48 and 72 h, respectively. The highest bioactivity of ChAgG was detected using 200 µg/mL, at 48 and 72 h, where the % of cell growth was 27 ± 0.5 and 28 ± 1% respectively. Despite good results, our ChAgG samples decrease importantly (ANOVA P > 0.05) the % growth of *E. coli* (ATCC 25922) but not as high as the gentamicin which was used as a positive control, which reduce the ability of cell proliferation (~ 22%), but optimization of our formulation still can be made increasing its bioactivity.

**Table 1:** Biological characterization of ChAgG samples in pathogen bacteria and cell lines.

ChAgG (µg/mL)	*Escherichia coli* (ATCC 25,922)			*Staphylococcus aureus* (ATCC 25,923)		
	24 h	48 h	72 h	24 h	48 h	72 h
400	37 ± 2	28 ± 0.7	36 ± 3	84 ± 13	81 ± 0.8	78 ± 4
200	58 ± 4	27 ± 0.5	28 ± 1	83 ± 9	75 ± 3	84 ± 6
100	35 ± 3	49 ± 1	37 ± 5	89 ± 4	93 ± 16	94 ± 2
50	87 ± 0.5	82 ± 4	84 ± 12	92 ± 15	90 ± 18	83 ± 1
25	71 ± 3	63 ± 7	68 ± 7	95 ± 14	93 ± 4	88 ± 4
12.5	74 ± 1	73 ± 1	65 ± 3	98 ± 24	89 ± 7	75 ± 3
6.25	49 ± 3	54 ± 0.2	62 ± 5	112 ± 10	105 ± 12	100 ± 1
3.125	48 ± 0.7	46 ± 0.7	38 ± 0.3	105 ± 5	98 ± 8	102 ± 2
Negative control	100 ± 1	100 ± 5	100 ± 2	100 ± 2	100 ± 3	100 ± 5
Positive control	26 ± 0.7	29 ± 4	22 ± 0.6	35 ± 0.8	28 ± 1	20 ± 0.6

All data are expressed in % of growth calculated using the growth of negative control (cell growth without any stimuli). Experiments were done in triplicate.

The selected bacterial strains in this study can cause serious skin damage, for example, *Staphylococcus aureus* is associated with skin and soft tissue infections in ambulatory care^56^, while *Escherichia coli* can be isolated from surgical and traumatic wounds, foot ulcers, and decubitus^3^. Likewise, each of them are widely studied bacterial models, so they can be used for comparative purposes and likewise as representatives in their cell walls, being *E. coli* Gram-negative and *S. aureus* Gram-positive.

The results showed that ChAgG compound has better behavior against *Escherichia coli* (ATCC 25922), however, against *S. aureus* (ATCC 25923) was not as effective, a factor to consider is the multiple drugs resistance phenotype of the *S. aureus* bacteria (ATCC 25923). That makes it one of the most difficult pathogenic bacteria to treat^57^, so one of the reasons to which can be attributed these results may be the low percentage of the antibacterial compound used. Hence, Ch-functionalized compound show higher efficacy for *E. coli* (ATCC 25922) than for *S. aureus* (ATCC 25923) due to the difference in their cell walls in a study published by Dutta et al., in which it has also been shown that the antibacterial activity of chitosan incorporated, either with silver ions (Ag) or nanocrystals, is greater than the activity of each component individually^58^.

It has also been shown that the potential of G-based bacterial surfaces can be enhanced by using silver, zinc, and iron nanocrystals. Our ChAgG sample, as already mentioned, showed greater efficacy against *E. coli* (ATCC 25922)*.* Similar results were reported by Gu et al., where it was evaluated the antibacterial properties of graphene supplemented with chitosan, confirming that the inhibition effect was stronger and dependent on the concentration of said components^59^.

### MTT cell viability test

The MTT assay was performed to measure the metabolic activity of cells and estimated through the ‘color-change’ phenomenon from yellow-colored tetrazolium salt, MTT 3-(4,5-dimethylthiazol-2-yl)-2,5-diphenyltetrazolium bromide to purple-colored formazan^60^. In this case, it was carried out with mouse fibroblasts L-929 where after 24 h of incubation it was observed that there was no difference between cell proliferation when compared to control (normal cell growth).

The proliferation of the L-929 cultures showed that the ChAgG compound decreased by ~ 21%. A slight pattern can be observed where between higher the concentration of the ChAgG compound higher the alteration of cell growth (%). Nevertheless, at 100 µg/mL was observed the highest cytotoxicity with a ~ 16% of cell growth reduction, and at 3.125 µg/mL a slight increment of 2% was observed. Almost no alteration (ANOVA P > 0.05) in cell growth was observed between concentrations of 50–3.125 µg/mL. Despite that, all these positive or negative alterations do not represent a major change in cell behavior.

**Figure 6:**
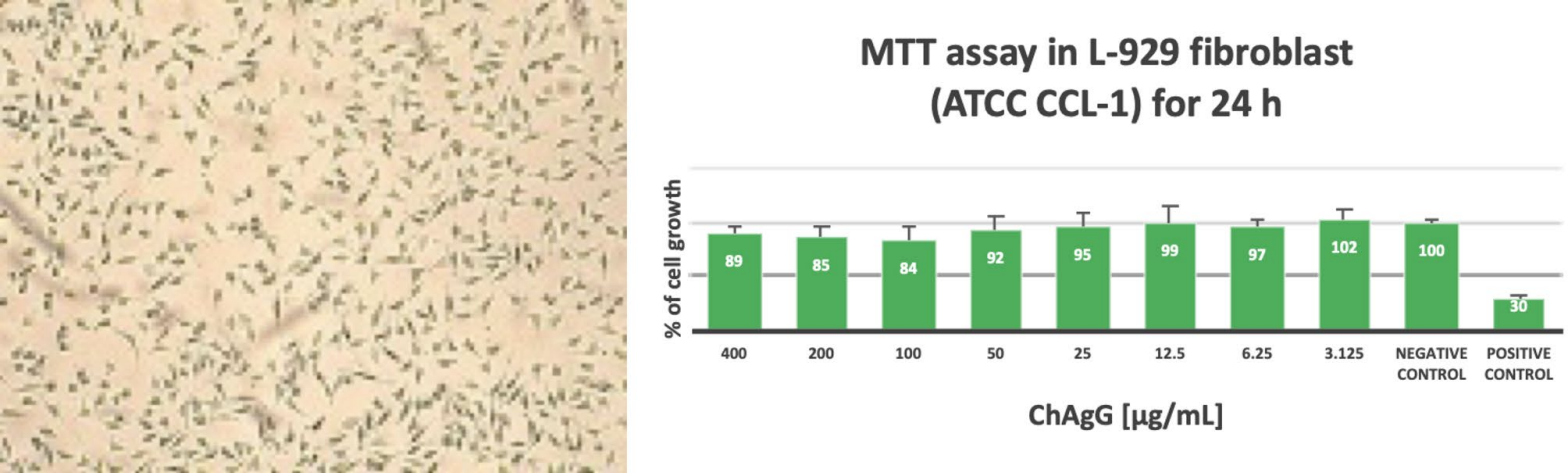
MTT cell viability test of PCL/PVP/ChAgG fibers. Experiments were done by triplicated. Average and standard deviation are presented.

When performing an analysis of variance (ANOVA P > 0.05) comparing the unexposed samples with those exposed for 24 h, it was observed that there was no significant difference in the normal growth of the L929 fibroblasts. Although, in the analysis performed comparing the several concentrations between them, they did not show a significant difference to decide which of the samples is better, however, by showing that there was no significant alteration in the normal growth of L929 fibroblasts after 24 h, we assume that the ChAgG compound is not toxic to the human body, but this biocompatibility will depend of concentration, where at highest concentration cytotoxicity can be increased (Fig. 6).

L929 cells were used in the present study because they can be easily cultured in a reproducible manner, and also this cell line is widely used for preliminary cytotoxicity evaluation for a wide range of biomaterials because of its easy proliferation and adherence on most of the biomaterial surface^61^.

About the reported cytotoxicity of chitosan, silver nanocrystal, and graphene in the presence of L929 cells. Gao et al. discussed the good biocompatibility and improved cellular attachment of L929 fibroblast cells^62^. Moreover, Toullec et al. demonstrated that the presence of pure chitosan electrospun scaffolds does not provoke any alteration on L929 cell proliferation^63^.

In the case of the cytotoxicity of graphene, Lasocka et al.^64^ evaluated the cytotoxicity of pristine graphene monolayer using the murine fibroblast L929 cell line. In that study, graphene demonstrated no cytotoxicity in L929 fibroblast and increased cell adhesion and proliferation within 24 h of culture. The authors discussed the potential capacity of graphene for the recovery of damaged subcutaneous connective tissue after injury^64^.

Finally, AgNCs cytotoxicity on L929 cells is concentration-depended, hence, a higher concentration of AgNCs presence lower cell proliferation, this phenomenon is observed in our ChAgG samples as well as the study of Składanowski et al., where the patterns of the same results are observed between both studies^65^. Hence ChAgG sample’s biocompatibility will depend on the concentration of AgNCs present in the final formulation.

### General properties of the ChAgG nanocomposites

The objective of this study is to report a sequential set of characterization techniques that helps in the decision making of the experimental steps for the antimicrobial ChAgG nanocomposite synthesis. From the selection of a natural source for AgNCs production to the demonstration of chemical interaction between chitosan, graphene oxide, and the resulting AgNCs. Table 2 summarizes the series of techniques used to follow the careful synthesis of the ChAgG nanocomposite finalizing with the demonstration of the potential used in biomedical applications.

**Table 2:** General sequence and results obtained of the synthesis of the ChAgG nanocomposites.

1. TGA	2. XRD	3. SEM
Higher residual mass (~ 5%) obtained from garlic source than onion in AgNCs synthesis	Crystalline cubic structure of AgNCs A5’s sample showed the highest crystallinity	384.6** ± **168.2 nm conglomerate’s crystals size

4. HRTEM	5. Raman	6. XPS
5.6** ± **1.5 nm particle size of AgNCs	Evidence is given in the oxidation of graphene by conventional microwaves and the generation of defects by the generation of the ChAgG nanocomposite	AgNCs present in the ChAgG nanocomposite and it is interacting with oxygen atom of the GrOx and chitosan

7. Antibacterial bioactivity		8. In vitro biocompatibility
Highest bioactivity was observed with just 3.125 µg/mL of the ChAgG nanocomposite reducing bacterial population about ~ 52, 54, and 62% at 24, 48 and 72 h, respectively		Almost no alteration (ANOVA P > 0.05) in cell growth was observed between concentrations of 50–3.125 µg/mL

From an initial screening of 10 samples, 5 using garlic as a source (A1–A5) and 5 using onion (C1–C5), were tested and the residual mass percentage (Ag presence) was calculated selecting two samples from the garlic source. Secondly, the cubic structure of the resulting AgNCs was characterized with XRD and general morphologies, and the size of the AgNCs nanocrystals and agglomerated were tested with HRTEM, and SEM, respectively. Then, Raman and XPS demonstrated the chemical interaction between chitosan, graphene oxide, and AgNC. Finally, bioactivity and biocompatibility were successfully demonstrated.

### Future perspectives

Traditional wound dressings e.g., bandages and gauzes, although highly absorbent and effective for drying mild, exuding wounds, require a regular application, which therefore can cause pain upon dressing change. In addition, they have poor adhesion properties and cannot provide enough drainage for the wound. In this regard, the normalization of the healing process in chronic wounds is an extremely urgent task of public health and requires the creation and implementation of affordable dressings for patients with chronic wounds^66^.

Despite all, the main issue to solve is bacterial infections in chronic wounds. Electrospun nanofibers offer a promising solution to the management of wound healing, and numerous options are available to load antibacterial compounds onto the nanofiber webs^67^.

In our previous work, it was demonstrated that PCL/PVP 85:15 and PCL/PVP loaded with Ag-Si/Al_2_O_3_ fibrous scaffolds are non-toxic, flexible, with antimicrobial activity (Gram-negative bacteria *Pseudomona aeruginosa* and *Escherichia coli*, Gram-positive *Staphylococcus aureus* and the fungus *Candida albicans*), and can absorb and retain water. These components were effectively to be used as wound dressing generating effective wound healing. The biocompatibility was evaluated in HFF-1 fibroblast after 24 h of exposure^68,69^.

The blend of chitosan, silver nanocrystals, and graphene has been previously investigated demonstrating a great capacity for biomedical applications^12^. Chitosan is biodegradable, non-toxic, and promotes wound healing^13^, silver nanocrystals (AgNCs) give antimicrobial properties and reduce inflammation^14^, and graphene also confers self-antibacterial property and antibacterial properties in combination with different substances, excellent mechanical properties, conductivity, and biocompatibility^7^.

Hence, our research group will prepare and characterize PCL/PVP-ChAgG fibers and test their morphological, physicochemical, and mechanical properties of the fibers to create potential wound dressing for further biological studies.

## Conclusions

Bioactive wound dressings to treat superficial skin or burn wounds that generate high exudation are desired. One of the important issues in these treatments is microbial contamination and dressing attachment to the affected zones, complicating skin healing and recovery. Our work proposed an antimicrobial nanocomposite (ChAgG) that includes chitosan, silver nanocrystal, and graphene. These components will give the final wound dressing a potential bioactive characteristic. The characterization arrays which include TGA, XPS, Raman, HRTEM, and TEM demonstrated the successful production of silver crystal graphene oxidation and the blending of the components. TGA was useful to decide whether silver nanocrystals will be prepared from garlic. X-ray diffraction demonstrated that A5’s sample has a higher crystallinity with respect to A1’s sample, therefore, being A5 the ideal preparation for the synthesis of the ChAgG nanocomposite. HRTEM and TEM demonstrated the silver nanocrystal formation with cubic structures with sizes of 2–10 nm, Moreover, the Raman study provides evidence that chitosan plays a binder role between graphene and silver nanocrystals. XPS demonstrated the successful preparation of the whole ChAgG nanocomposite. Finally, it was demonstrated that the ChAgG composite possesses better antimicrobial bioactivity against *Escherichia coli* rather than *Staphylococcus aureus* and the final composite does not provoke important cytotoxicity to L-929 fibroblast cells validating its biocompatibility in vitro*.* For future work, the ChAgG nanocomposite will be used to functionalize electrospun nanofibers to create bioactive antimicrobial wound dressings.

## Supplementary Information

Supplementary Information.

## Data Availability

The data used to support the findings of this study are included within the article and they are available from the corresponding author upon request.
