# Supplementary material for: Antibacterial nanocomposite of chitosan/silver nanocrystals/graphene oxide (ChAgG) development for its potential use in bioactive wound dressings
Source: Sci Rep. 2023 Jun 23;13:10234. doi: 10.1038/s41598-023-29015-y (PMC10290094; doi:10.1038/s41598-023-29015-y)
Supplement: Supplementary file 1 — Supplementary Information. [file 41598_2023_29015_MOESM1_ESM.docx]

Supplementary Information

**Antibacterial nanocomposite of chitosan/silver nanocrystals/graphene oxide (ChAgG) development for its potential use in bioactive wound dressings**

Yoxkin Estévez-Martínez^1*^, Rubí Vázquez Mora^1^, Yesica Itzel Méndez Ramírez^1^, Elizabeth Chavira-Martínez^2^, Rafael Huirache-Acuña^3^, Jorge Noé Díaz-de-León-Hernández^4^, Luis Jesús Villarreal Gómez^5,6*^

^1^Tecnológico Nacional de México, Campús Acatlán de Osorio, Carretera Acatlán - San Juan Ixcaquistla kilómetro 5.5, Del Maestro, Unidad Tecnológica Acatlán, Acatlán, Puebla. 74949 México.

^2^Instituto de Investigaciones en Materiales, Universidad Nacional Autónoma de México, Circuito Escolar S/N, Ciudad Universitaria, Ciudad de México, 04510 México.

^3^Facultad de Ingeniería Química, Universidad Michoacana de San Nicolás de Hidalgo, Morelia, Michoacán, 58060, Mexico

^4^Centro de Nanociencias y Nanotecnología Universidad Nacional Autónoma de México, Carretera Tijuana-Ensenada, Km. 107, Ensenada, Baja California, 22860, Mexico

^5^Facultad de Ciencias de la Ingeniería y Tecnología, Universidad Autónoma de Baja California, Blvd. Universitario #1000. Unidad Valle de las Palmas. Tijuana, Baja. CP., Tijuana, Baja California, 21500 México

^6^Facultad de Ciencias Química e Ingeniería, Universidad Autónoma de Baja California, Universidad #14418, UABC, Parque Internacional Industrial Tijuana, , Tijuana, Baja California, 22424 México

*Corresponding author: yoxkin@gmail.com and luis.villarreal@uabc.edu.mx.


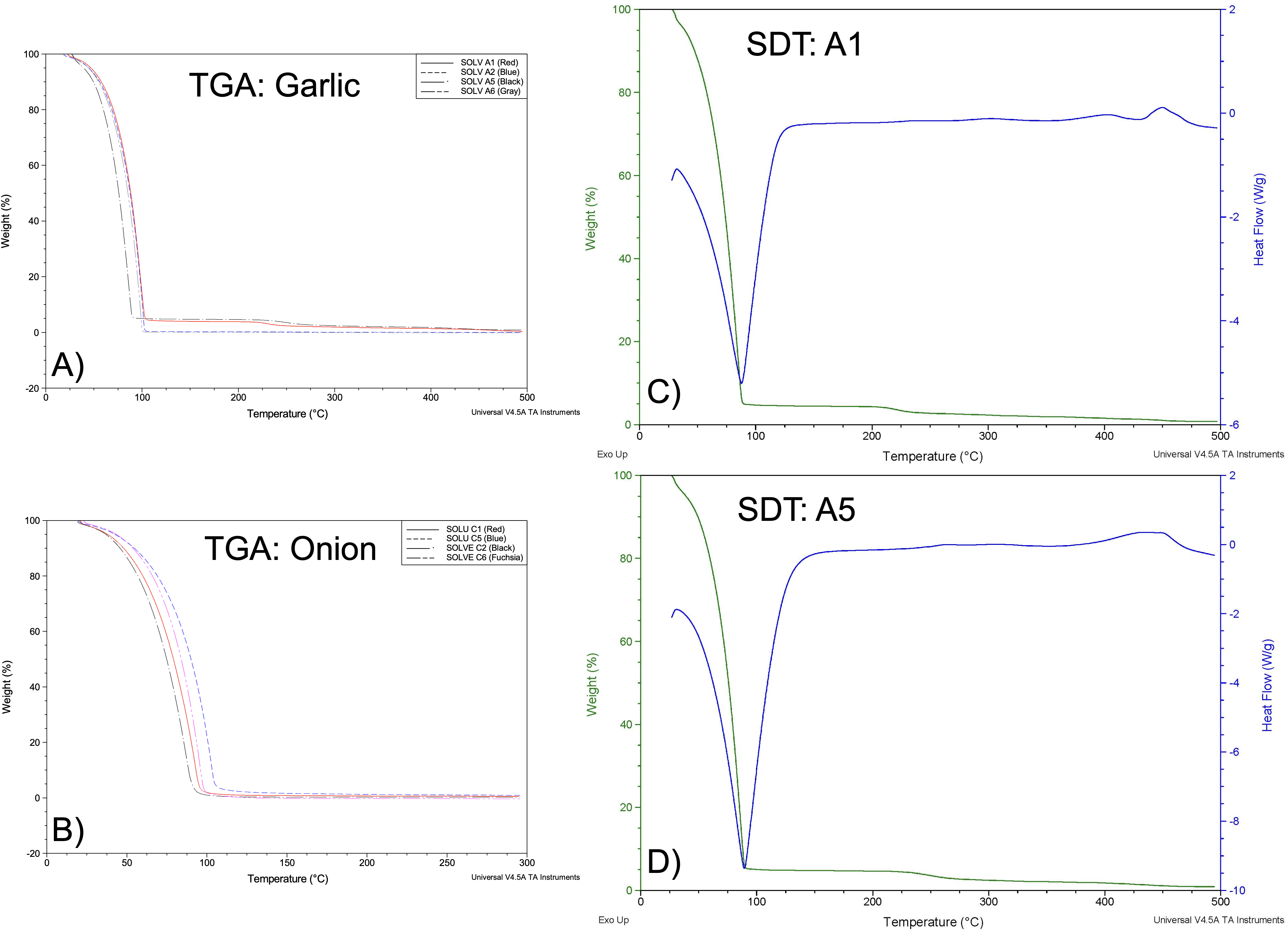


Figure-S1. Thermograms of garlic samples (A), onion samples (B) and, Simultaneous Differential Scanning Calorimetry (SDT) of A1 (C) and A5 (D) samples, with silver nanocrystals as precursors.


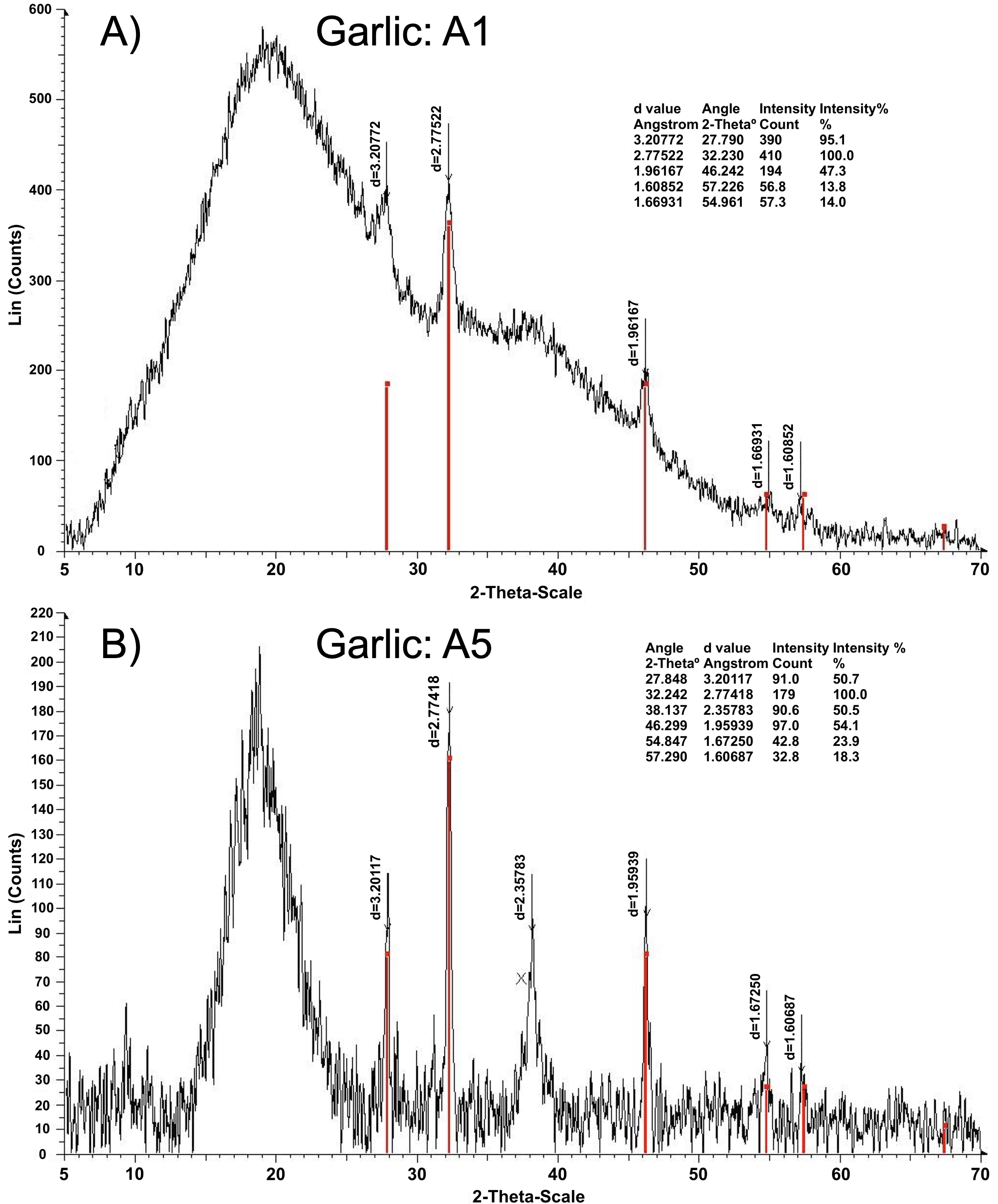


Figure-S2. XRD spectrum for the samples corresponding to garlic (A1, A5).


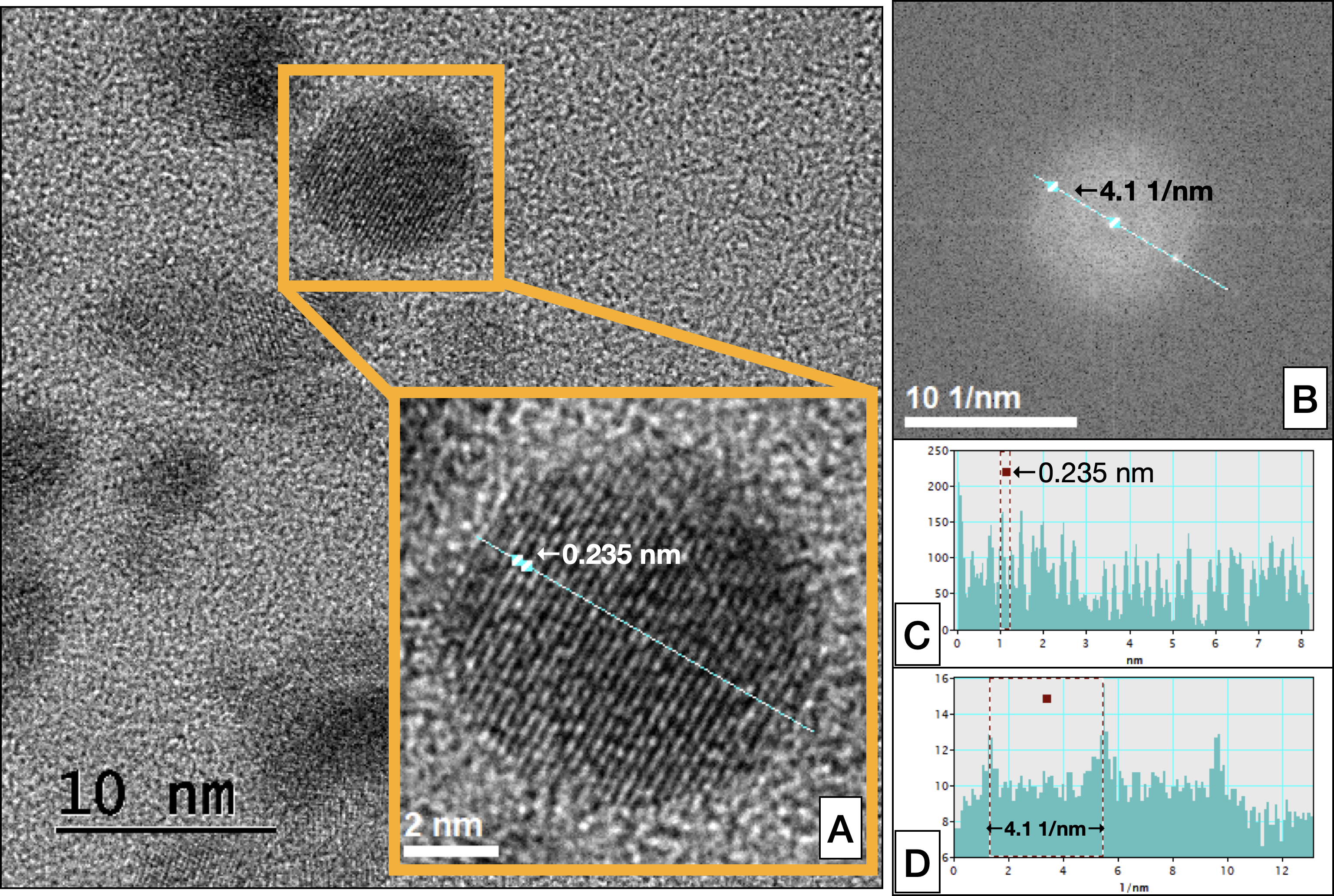


Figure-S3. A) d-spacing and lattice Fringes of AgNCs. B) HR-TEM SAED patterns of indexed of AgNCs. C) DLS histogram of d-spacing and lattice Fringes of AgNCs. D) DLS histogram of HR-TEM SAED patterns of indexed of AgNCs.


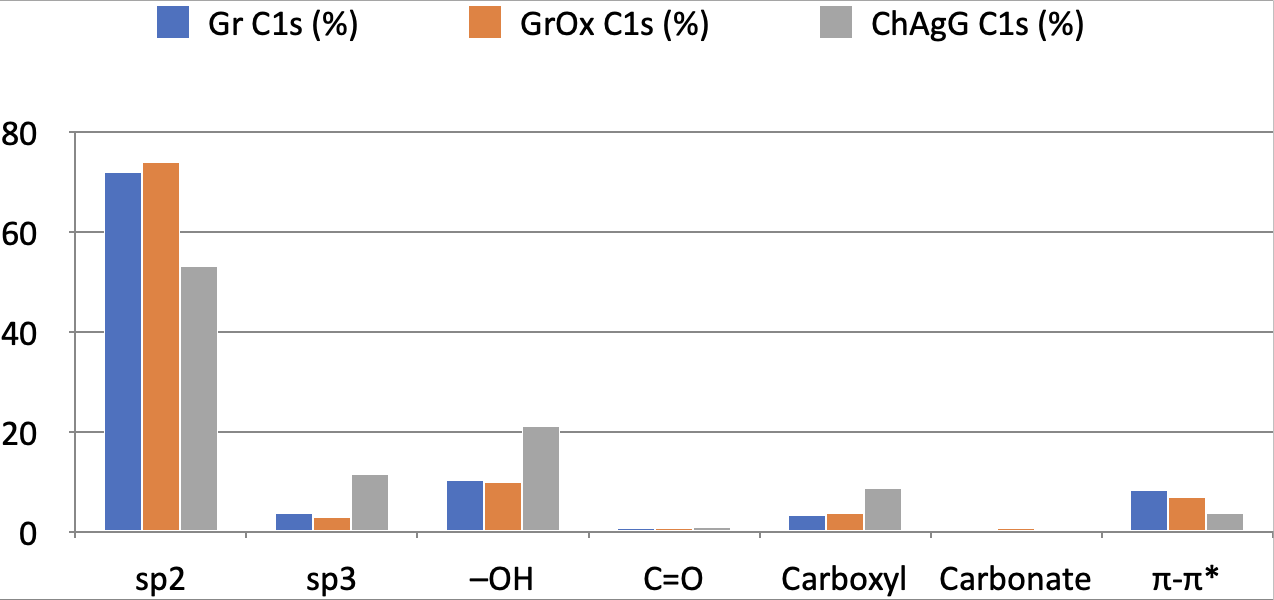


Figure-S4. XPS Analysis of C1s of the three samples (Gr, GrOx and ChAgG).


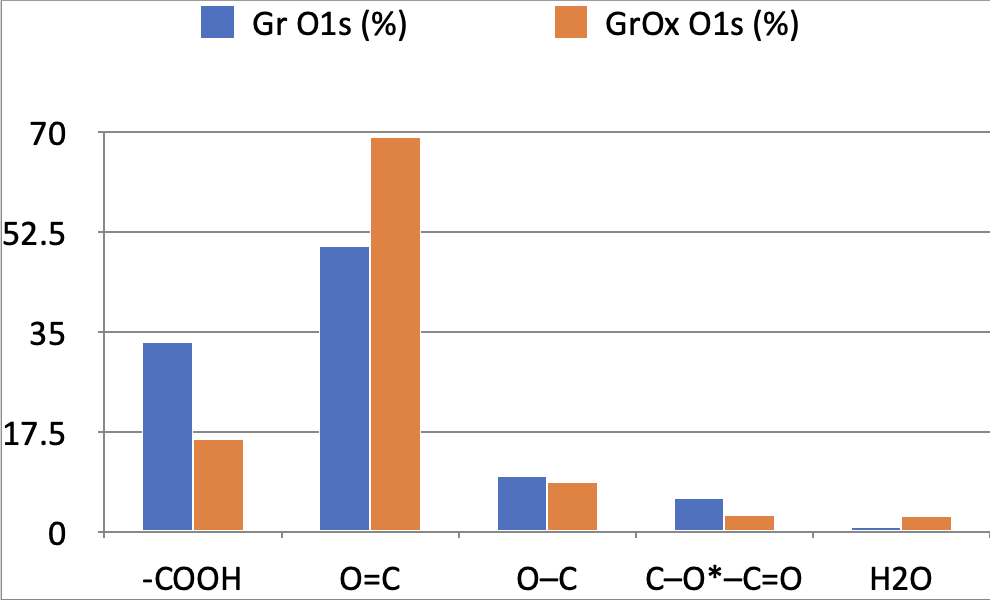


Figure-S5. XPS Analysis of O1s of the graphene samples (Gr and GrOx).


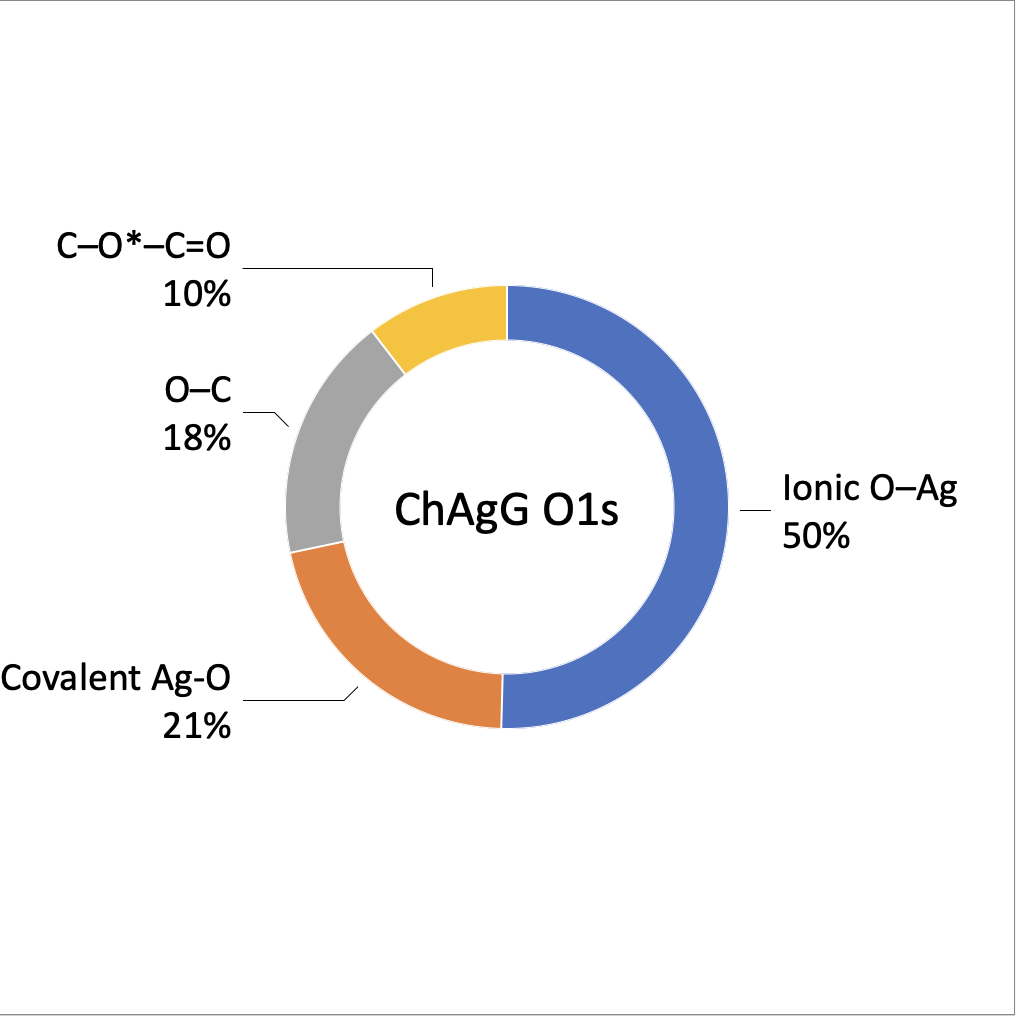


Figure-S6. XPS Analysis of O1s of the nanocomposite sample (ChAgG).
